# Increases in intravenous magnesium use among hospitalized patients: an institution cross-sectional experience

**DOI:** 10.1186/s40697-015-0056-7

**Published:** 2015-06-13

**Authors:** Bryce A. Kiberd, Karthik K. Tennankore, Christopher J. A. Daley

**Affiliations:** Department of Medicine, Dalhousie University, Queen Elizabeth II Health Sciences-VG Site, Room 5082 Dickson Building, 5820 University Avenue, Halifax, Nova Scotia B3H 1V8 Canada; Department of Medicine, Dalhousie University, Queen Elizabeth II Health Sciences-VG Site, Room 5070 Dickson Building, 5820 University Avenue, Halifax, Nova Scotia B3H 1V8 Canada; Pharm D. College of Pharmacy, Dalhousie University, Queen Elizabeth II Health Sciences-VG Site, Room 2043, 1276 South Park Street, Halifax, Nova Scotia B3H 2Y9 Canada

**Keywords:** Hypomagnesemia, Magnesium treatment, Magnesium, In-hospital, Drug shortage

## Abstract

**Background:**

Among hospitalized patients, indications for the measurement of magnesium levels and treatment of hypomagnesemia with intravenous magnesium are not well defined. Recently, there have been reports of worldwide shortages of intravenous magnesium sulphate.

**Objective:**

To examine secular trends in the administration of intravenous magnesium on hospital wards at a tertiary care institution. The secondary objective is to identify factors associated with magnesium use among admitted patients.

**Methods:**

Retrospective cross-section review of hospitalized patients at a single Canadian tertiary care center. Utilization of non-parental nutrition intravenous magnesium from 2003 to 2013 stratified by hospital ward was examined. In addition, patient level data from select wards (including medical and surgical services) was examined at early and more recent time period (4/2006 versus 4/2013).

**Results:**

Among the 248,329 hospitalized patients, intravenous magnesium use increased by 2.86 fold from 2003 to 2013. Not all wards had an increase whereas some had nearly a 10 fold increase in use. In the sample (*n* = 769), (adjusting for admission magnesium level, presence of an indication for intravenous magnesium, ward location, comorbidity and demographics) intravenous magnesium administration was higher (25.8 % versus 5.5 %) in 2013 versus 2006 (OR 13.91 (95 % CI, 6.21-31.17, *p* < 0.001). Despite this increase in intravenous magnesium administration, <3 % of patients were admitted on oral magnesium in 2006 and 2013. For patients receiving intravenous magnesium only a minority were discharged on oral therapy despite low levels.

**Conclusions:**

This center has witnessed a considerable increase in the use of in-hospital intravenous magnesium over the last 6 years that cannot be explained for by medical indications. The risks and benefits of this therapy deserve further study. If this change in practice is representative of other North American hospitals, it may be responsible for recent drug shortages.

## Abrégé

**Contexte:**

Les indications concernant la mesure des niveaux sanguins de magnésium et le traitement de l’hypomagnésémie par infusion intraveineuse de magnésium chez les patients hospitalisés sont mal définies. On a récemment rapporté des pénuries à l’échelle mondiale de sulfate de magnésium destiné à l’infusion intraveineuse.

**Objectifs:**

D’une part, examiner les tendances séculaires en matière d’administration de magnésium par voie intraveineuse dans divers services hospitaliers d’un centre de soins tertiaires. D’autre part, déterminer les facteurs associés à l’utilisation du magnésium chez les patients hospitalisés.

**Méthodes:**

Étude transversale et rétrospective auprès des patients hospitalisés dans un seul centre canadien de soins tertiaire. On a étudié un échantillon qui témoigne du recours à l’administration intraveineuse de magnésium, de 2003 à 2013, en stratifiant par service hospitalier.

**Résultats:**

Chez les 248 329 patients hospitalisés, le recours à l’infusion de magnésium par voie intraveineuse a augmenté de 2,86 fois entre 2003 et 2013. Dans certains services, aucune augmentation n’a été notée alors que dans d’autres, l’utilisation a augmenté de près de 10 fois. Dans l’échantillon (*n* = 769), (ajusté selon le taux de magnésium administré, la présence d’une indication nécessitant une infusion de magnésium, l’emplacement des services, la comorbidité et les données démographiques), l’administration de magnésium par voie intraveineuse était plus fréquente (25,8 % par rapport à 5,5 %) en 2013 qu’en 2006 (RIA = 13,91 (IC, 95 %, 6,21-31,17, *p* < 0,001). Malgré cette augmentation de l’utilisation du magnésium par voie intraveineuse, <3 % des patients avaient été admis pour administration orale de magnésium en 2006 et en 2013. Parmi les patients qui ne recevaient le magnésium que par voie intraveineuse, seule une minorité avait reçu son congé avec une thérapie par voie orale, en dépit des faibles taux.

**Conclusion:**

Nous avons observé une augmentation considérable dans l’utilisation de l’infusion de magnésium par voie intraveineuse en milieu hospitalier au cours des 6 dernières années, ce qui n’est pas justifié par des indications d’ordre médical. Les avantages et les risques que comporte cette thérapie méritent une étude approfondie. Ce changement de pratique, s’il s’observe dans d’autres hôpitaux nord-américains, pourrait expliquer les récentes pénuries de médicaments.

## What was known before

There have been recent shortages of magnesium sulphate for intravenous use in North America from increased demand. The reasons for this increase in utilization are unclear given the lack of evidence for its use in clinical randomized trials outside of the prevention of pre-eclampsia.

## What this adds

Intravenous magnesium sulphate use has increased nearly 3 fold in this tertiary Canadian hospital. The increase cannot be explained by patient characteristics and likely represents a change in practice. Few patients were discharged on oral magnesium supplements despite low levels at discharge and follow up. Evidence based guidelines for the measurement and treatment of low magnesium are needed.

## Background

Magnesium is an essential nutrient for humans. Magnesium is the second most abundant intracellular cation and is a cofactor for many enzymes that are required for healthy cell function. Profound deficiency is associated with weakness, tetanus and cardiac arrhythmias [1, 2]. The prevalence of hypomagnesaemia is variable and depends on the laboratory definition of a low normal level. In hospitalized patients, as high as 10-15 % have hypomagnesaemia and this increases to >50 % in intensive care units [1].

Hypomagnesemia has been associated with several adverse outcomes and medical conditions. Low total magnesium levels among intensive care unit patients are associated with higher rates of mortality [3–5]. Hypomagnesaemia and low magnesium intake by dietary recall are associated with diabetes mellitus, cardiovascular disease including stroke in some but not all community based cohort studies [6–9]. While hypomagnesemia may be associated with poor outcomes, the benefits of correcting hypomagnesemia are not clear in all conditions. In some but not all studies, magnesium supplementation is associated with improved blood pressure, lipid and glucose control [2, 10–12]. There is evidence that therapeutic doses of magnesium might improve blood flow and reduce reperfusion injury [13]. Intravenous magnesium is very effective in pre-eclampsia [14]. Intravenous magnesium is helpful in some patients with ventricular arrhythmias [13]. There are also a large number of randomized controlled studies examining the role of intravenous magnesium administration to improve outcomes associated with myocardial infarction, atrial fibrillation, subarachnoid hemorrhage, traumatic brain injury, and in the treatment of acute asthma [15–19]. However, several of the meta-analyses and systematic reviews of these trials have been contradictory or suggest that alternative therapies are superior [20–22].

In 2011 [23], there were world-wide shortages of many generic medications, leading President Obama to charge the Federal Drug Administration with ensuring adequate supply of important medications for the US population. Intravenous magnesium sulphate was cited as one of the medications being in short supply [24]. For magnesium sulphate the shortage appeared to be created by increased demand.

Therefore, the objective of this descriptive study was to examine intravenous magnesium use in a large tertiary Canadian hospital over the last 10 years. Furthermore, we sought to determine factors associated with administration of magnesium in a restricted cohort of patients during this time period. We hypothesized that there would be a secular trend to higher use of intravenous magnesium. The primary outcome would be a quantification of use with secondary outcomes of interest to determine which hospital units, which patients and for what indications. Additionally we tested the hypothesis that the increase would be independent patient disease and co-morbidity.

## Methods

### Derivation of cohort

Magnesium sulphate use was determined from pharmacy records at Capital Health (New Halifax Infirmary and Victoria General sites). This institution is a tertiary care center for Nova Scotia (population approximately 960,000) but also provides specialized care for the other Atlantic Provinces. Since magnesium sulphate is not unit dosed, floor consumption was estimated from quarterly use of existing floor stock records. Fiscal year data was available from 2003 to the last quarter of 2013. The focus of this analysis was intravenous magnesium use on the in-hospital medical units (as opposed to operating rooms, emergency and out-patients clinics) and did not include magnesium used in parental nutrition solutions. The units of interest included all intensive care units (cardiac care unit, cardiac surgery, and two medical-surgical intensive care units), surgical floors (general (9A and 9B), urology (5B), surgical oncology (5A), thoracic (6A), orthopedics (7.2), neurosurgery (7.3), vascular (4.1 and 7.1) medical floors [general (8.2), neurology (8.1), hematology (8B), cardiology (6.2 and 6.4)] and mixed floors (6B and 8.3).

Since this institution level data does not identify which patients received intravenous magnesium and what the indications were, a detailed chart review of patients from five floors (cardiology (6.2), cardiac care unit (6.4), general surgery (9A and 9B), mixed surgery/nephrology (6B), general medicine (8.2 and 8.4)) was also undertaken. These floors were identified to have considerable increases in magnesium use over the time period.

### Derivation of restricted cohort

All consecutive patients discharged (including death) in April 2006 and April 2013 were reviewed to eliminate selection bias. The year 2006 was chosen since this was the onset of the increase in use of intravenous magnesium and this was the year that hospital records were made electronic. April 2013 was selected as this allowed 6 months of follow up at the time of data collection. Patients discharged within 24 h, those who had no blood work during the admission and those who were readmitted within the month were excluded.

### Outcome and potential confounders

The primary outcome of the analysis on this restricted cohort was intravenous magnesium use (yes/no). To identify factors associated with magnesium use, data was collected on discharge diagnosis, whether patients were admitted through emergency, whether their stay included time in an intensive care unit, and whether they received surgery. Information on patient co-morbidity was collected including ischemic heart disease, congestive heart failure, atrial fibrillation, chronic kidney disease, hypertension, diabetes mellitus, liver disease, alcohol abuse (as defined in the records), cancer and chronic obstructive pulmonary disease. Comorbid conditions were based on documentation through electronic patient records. All encounters including inpatient and outpatient were reviewed. Hospital admission laboratory data including serum creatinine, potassium, hemoglobin, albumin, calcium and magnesium was collected. Magnesium, albumin, calcium, and potassium levels were also collected at the time of intravenous magnesium use if available. Estimated glomerular filtration rate (GFR) was measured by the four variable Modified Diet in Renal Disease (MDRD) formula [25]. The charts were reviewed to determine if patients were on oral magnesium, calcineurin inhibitors, proton pump inhibitors or non-potassium sparing diuretics. For patients with a history of cancer the chart was reviewed to look for evidence of prior cisplatin administration. Admission and in-hospital EKG reports were examined for a diagnosis of arrhythmia (including atrial fibrillation, ventricular tachycardia or fibrillation, and long corrected QT interval-as defined by the cardiologist report). In patients receiving intravenous magnesium follow up blood work was reviewed to determine if serum magnesium levels had been done after discharge within a 6-month period. In addition to patient factors, each patient record was reviewed to determine the indication for intravenous magnesium administration. Since there are no written evidence based guidelines for intravenous magnesium administration, prior to data analysis, the authors after a review of the literature agreed indications should include severely low levels (magnesium ≤0.5 mmol/L), or a low level (0.5 to 0.74 mmol/L) in patients with long corrected QT or other arrhythmia, low potassium (≤3.0 mmol/L), low corrected calcium (≤2.0 mmol/L), neuromuscular problems or ongoing gastrointestinal loss (diarrhea, vomiting, post operative ileus or bowel obstruction) [1, 26].

Serum magnesium was measured on the hospital’s Synchron DxC (Beckman Coulter Inc., CA). The reported normal range is 0.74 to 1.03 mmol/L. The co-efficient of variation is 3.8 % in plasma. There were no changes in methodology over the 10-year period. A corrected magnesium and calcium was calculated if an albumin level was available. The formulas were as follows: corrected magnesium [mmol/L] = magnesium [mmol/L] + 0.005*(40-albumin [g/L]) and corrected calcium [mmol/L] = Calcium [mmol/L] + 0.025* (40-albumin [g/L]) respectively [27, 28].

### Statistical analysis

Data for magnesium use in the institution was collected by fiscal year. Baseline characteristics of patients from 2006 and 2013 were described as means ± standard deviation (SD) for continuous normally distributed variables, median and interquartile range for non-normally distributed variables and number and percentage for categorical variables. Statistical tests of comparison were performed using independent t-tests, the Rank Sum test and Fisher’s Exact test, accordingly. Multivariable nested logistic regression models were used to determine if there was an independent association between era (2013 versus 2006) and use of intravenous magnesium among those patients with available magnesium laboratory measurements. Variables selected for inclusion in the multivariable models were performed *a priori* and included demographics, co-morbidities, medications associated with hypomagnesaemia, presence or absence of a low serum magnesium level at hospital admission, and whether or not there were indications to give magnesium intravenously based on rationale outlined above. For all statistical analyses a 2 sided P value <0.05 was considered statistically significant. All analyses were performed using Stata IC version 12.0 (StataCorp LP, College Station, TX). Research ethics approval to conduct this study was obtained from Capital Health Research Ethics Board.

## Results

**Fig. 1:**
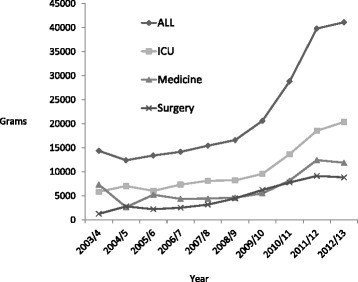
All Intravenous Magnesium Use on Hospital Wards 2003–2013. Patient Flow for the 2006 Cohort

**Fig. 2:**
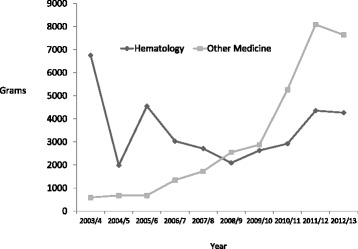
Intravenous Magnesium Use on Medical Wards 2003–2013. Patient Flow for the 2013 Cohort

**Fig. 3:**
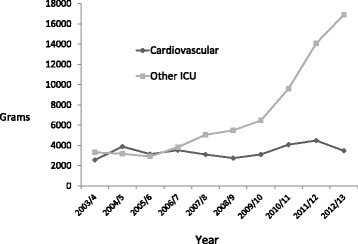
Intravenous Magnesium Use on Surgical Wards 2003–2013

Magnesium Use at The Hospital Level: Over the last seven years (2006–2013) of this ten-year review, there was a 2.86 fold increase in magnesium use on the hospital wards and intensive care units. Figure 1 shows the change in all, intensive care units, medical and surgical floors. Despite the increase in use there was variability between individual units. Hematology (including the bone marrow unit) was a heavy user of intravenous magnesium, whereas the other medicine floors combined witnessed a 12.9 fold increase in intravenous magnesium use (Fig. 2). There was a 6.9 fold increase in intravenous magnesium use on the surgical wards. The cardiovascular intensive care unit had a consistently high use whereas the two medical surgical intensive care units had a 5.1 fold increase in use (Fig. 3). Over this time period there was very little variation in the annual number of hospital cases, length of stay and in hospital mortality (Table 1).

**Table 1:** Hospital Activity 2003-2013

Fiscal Year	Admissions	Average Length of Stay	Deaths	Mortality Rate
2003/4	23,768	9.6	1266	5.32
2004/5	24,470	9.51	1348	5.51
2005/6	24,675	9.61	1226	4.97
2006/7	24,016	10.26	1261	5.25
2007/8	24,597	10.28	1225	4.98
2008/9	24,807	9.72	1198	4.83
2009/10	25,489	9.82	1228	4.82
2010/11	25,613	9.65	1231	4.81
2011/12	25,595	9.31	1140	4.45
2012/13	25,299	9.28	1273	5.03

Magnesium Use at the Patient Level in 2006 and 2013 Cohorts: The patient level analysis was consistent with the findings of an increase in magnesium use. Characteristics of the 2006 and the 2013 cohorts were similar with respect to age, sex, co-morbidity status and laboratory values (Table 2).

**Table 2:** Baseline Characteristics of All Patients from 2006 and 2013

Variable	2006 (*N* = 397)	2013 (*N* = 372)	P Value
Age	61 ± 17	63 ± 15	0.153
Male Sex n (%)	228 (57)	211 (57)	0.884
Location			<0.001
Cardiology	126 (32)	122 (33)	
Coronary Care Unit	38 (10)	35 (9)	
Medical/Surgical Ward	29 (7)	62 (17)	
General Surgery	136 (34)	75 (20)	
Internal Medicine	68 (17)	78 (21)	
Medical Co-morbidities			
Diabetes Mellitus	112 (28)	114 (31)	0.477
Hypertension	221 (56)	214 (58)	0.611
Atrial Fibrillation	41 (10)	69 (19)	0.001
Congestive Heart Failure	63 (16)	53 (14)	0.547
Ischemic Heart Disease	134 (34)	102 (27)	0.061
Chronic Obstructive Lung Disease	55 (14)	61 (16)	0.364
Malignancy	69 (17)	69 (19)	0.707
Liver Disease	19 (5)	24 (6)	0.348
Chronic Kidney Disease	100 (25)	92 (25)	0.934
(GFR < 60 ml/min/1.73 m^2^)			
Alcohol Abuse	8 (2)	14 (4)	0.194
Gastrointestinal Symptoms	83 (21)	102 (27)	0.043
Surgery	96 (24)	87 (23)	0.865
Medications			
Calcineurin Inhibitor	9 (2)	13 (3)	0.388
Diuretics	125 (31)	121 (33)	0.758
Proton Pump Inhibitors	100 (25)	130 (35)	0.004
Oral Magnesium	9 (2)	11 (3)	0.652
Laboratory mean (SD)			
Calcium (Total) (*n* = 171/210)	2.17 (2.04-2.30)	2.10 (2.01-2.12)	0.002
Potassium (*n* = 396/369)	4.1 (3.8-4.5)	4.2 (3.8-4.5)	0.968
Hemoglobin (*n* = 396/371)	127 (111–143)	127 (108–140)	0.163
Albumin (*n* = 149/158)	30 (26–34)	30 (24–34)	0.649
MDRD GFR (396/266)	63 (42–81)	69 (45–88)	0.023
Magnesium (*N* = 173/233)	0.84 (0.75-0.92)	0.78 (0.71-0.86)	<0.001
Admission Magnesium Low (*n* = 173/233)	39 (23)	76 (33)	0.030

The average measured magnesium levels were lower in the 2013 cohort, whereas the frequency of testing, estimated MDRD GFR, use of proton pump inhibitors and prevalence of atrial fibrillation was higher compared to 2006. Very few patients (<3 %) were admitted on oral magnesium therapy in either era. Intravenous magnesium was administered to 22 of the 397 (5.5 %) patients and 96 of 371 (25.9 %) patients in the 2006 and 2013 cohorts respectively. Nearly 41 % (96/233) of 2013 patients with a magnesium level were given an intravenous dose in 2013 compared to 12 % (21/173) in 2006. All patients (12/12) spending time in the medical intensive care unit in 2013 were given intravenous magnesium compared to 67 % (8/12) in 2006. Excluding patients spending time in an intensive care unit, intravenous magnesium on medical and surgical floors increased 6.9 fold. In the fully adjusted model (Table 3) the odds ratio for intravenous magnesium use was 13.91 (95 % CI, 6.21-31.17, *p* < 0.001) for patients in 2013 compared to 2006.

**Table 3:** Association between era (2013 versus 2006) and administration of intravenous magnesium

Model	Odds Ratio [95 % CI]	P
Unadjusted	5.07 [3.00-8.58]	<0.001
Model 1^a^	5.09 [3.00-8.62]	<0.001
Model 1^b^	5.60 [3.25-9.66]	<0.001
Model 1^c^	5.60 [3.24-9.67]	<0.001
Model 1^d^	5.98 [3.33-10.74]	<0.001
Model 1^e^	9.24 [4.78-17.87]	<0.001
Model 1^f^	12.89 [6.23-26.68]	<0.001
Model 1^g^	13.91 [6.21-31.17]	<0.001

Adjusted for:

^a^age, gender

^b^factors in a) and comorbidities (ischemic heart disease, diabetes, atrial fibrillation, cancer, heart failure, chronic kidney disease)

^c^factors in b) and medications (diuretics, proton pump inhibitors)

^d^factors in c) and low admission magnesium

^e^factors in d) and admission ward location (cardiology, coronary care unit, medicine ward, surgical ward mixed medicine/surgery ward)

^f^factors in e) and cardiac indication for intravenous magnesium

^g^factors in e) and cardiac or other indication for intravenous magnesium

In the 2013 cohort, 39 % (37/96) had an indication for intravenous magnesium. These included ongoing loss and inability to take oral magnesium (15), EKG abnormalities (6), and electrolyte disturbances (16). However 25 % (24/96) had values in the normal range. Albumin was available in 70 of the 96 patients at the time. Of the 24 patients, 12 had a normal corrected magnesium and 12 had a level within the normal range (uncorrected). There were also significant differences in magnesium measured and treated for the same diagnosis. For example in the 2013 cohort, patients admitted to the coronary care unit with a myocardial infarction all had magnesium ordered and 64 % (18/27) received intravenous magnesium. Those admitted with a myocardial infarction to an alternative level cardiac unit were significantly less likely to have their magnesium measured (53 %, 16/30, *p* < 0.001) or receive intravenous magnesium (13 %, 4/30, *p* < 0.001).

Of the 96 patients in the 2013 cohort receiving intravenous magnesium, 90 had a repeated value at or near discharge (Table 4). Of these 28 patients (31 %) had a value that was less than 0.74 mmol/L that was not treated. Only 3 patients were discharged on oral magnesium and 2 of the 3 were on oral magnesium prior to admission. Only 25 patients had magnesium re-measured after discharge and of these 17 (68 %) were low with a mean level of 0.67 mmol/L.

**Table 4:** Follow up Magnesium Levels in Treated Patients: 2013 Cohort

Variable	Intravenous Magnesium Given (*N* = 96)
Discharge Magnesium PersistentlyLow (*N* = 90)	28 (31 %)
Magnesium Low After Discharge (*N* = 25)	17 (68 %)
Magnesium Level at Discharge mmol/L	0.80 (0.73-0.85)
>0.74	61
0.50-0.74	28
<0.50	0
Magnesium Level After Discharge mmol/L	0.67 (0.46-0.92)
>0.74	8
0.50-0.74	15
<0.50	2

## Discussion

The principal findings of this analysis are that there has been a significant increase in intravenous magnesium use in this hospital on most of the medical and surgical wards. The administration of intravenous magnesium may be unnecessary in a significant proportion of patients. There are no consistent indications for administration, and treatment behaviors are different among individual wards. The focus on treating hypomagnesaemia is during patients’ in-hospital stay. Many of the patients treated for hypomagnesaemia with intravenous magnesium left hospital with levels that were low or not measured.

This study shows that magnesium administration has increased over the last 7 years. The reasons behind this change in practice are independent of blood levels, case mix and co-morbidity. In patients with a measured magnesium level, blood levels were lower in the more recent cohort. However this difference might not have been seen if all patients were tested in 2006. Other unmeasured differences might be responsible for the increase in magnesium infusions. It is interesting to point out that proton pump inhibitor use is associated with hypomagnesemia [29] and hypomagnesaemia is associated with atrial fibrillation [30]. All three were more prevalent in the recent 2013 cohort. However there was no apparent connection that could be confirmed or refuted by this study.

Many of the patients especially those undergoing bowel surgery were identified to have hypomagnesemia and appropriately received intravenous magnesium. However the majority of other treated patients may not have required intravenous therapy. Correcting abnormal test results when possible is an easily defensible action when there is no contrary evidence that treatment would cause harm. There is however evidence of harm in the large trials using intravenous magnesium in patients with a myocardial infarction. Rates of bradycardia and hypotension were higher in the treated arms without any survival benefit [17]. However many of these subjects received high rates of infusion and may have developed hypermagnesemia. Paradoxically ionized hypermagnesaemia was associated with increased mortality in one study of patients in an intensive care unit [31]. The usual intravenous dose of 2 g (8 mmol) of magnesium sulphate administered over 1 h would transiently increase the magnesium levels to a hypermagnesemic state assuming the extracellular volume of distribution is 10–16 l of water.

There does not appear to be any evidence-based guidelines that inform indications for magnesium measurement. For example despite the evidence that hypomagnesemia is associated with atrial fibrillation and is useful to prevent post cardiac surgery atrial fibrillation, Canadian and US guidelines do not specifically recommend assessment of magnesium in the initial evaluation of atrial fibrillation [32, 33]. In addition the recommendations for treatment of deficiency are opinion based. For these reasons practice is likely to vary considerably within and between institutions. Some general medicine reference sites recommend treatment of asymptomatic hospitalized patients with intravenous magnesium for levels just below the normal range [26]. There seems to be less emphasis on oral replacement. Magnesium is an excellent laxative and may not be tolerated by many patients. However the increased intensity of measuring and treating hypomagnesaemia in hospitalized patients over the last 7 years has not been matched by treatment in outpatients. The medical aspects of low dietary magnesium intake and low serum levels in community populations have been studied extensively over the years [6–9]. Some but not all studies support magnesium supplementation or increased intake for blood pressure control, dyslipidemia, glucose metabolism, stroke prevention and dysrhythmia prevention [2, 6–12]. Oral magnesium supplementation in patients entering hospital is relatively low in our study and was not different between the 2006 and 2013 cohorts. If treatment of low magnesium is important, more attention is needed in the outpatient services.

This analysis may reflect a trend in health care, this being a desire to identify and aggressively treat many facets of an individual’s physiology while in hospital. This adds to the complexity and costs of care and may not result in any short or long term benefit. Intravenous magnesium is relatively inexpensive but the nursing and pharmacy time costs to administer the medication may be much higher.

There are several limitations to this study. This study was unable to determine whether intravenous supplementation would reduce in hospital mortality. A large randomized control trial would be necessary. We did not systematically examine all patients over the 10-year time period. However it is not clear a larger study would change the conclusions of this report, given the findings of the institutional analysis. It would also be interesting to know if this is a pattern seen in other centers in North America. The Federal Drug Administration website as of July 2014 continues to identify a shortage of intravenous magnesium sulphate from increased demand [34]. To date shortages in Canada have not been as acute [35]. With increasing demand, shortages in Canada might develop. Finally, while this study identifies an increase in administration of intravenous magnesium, the reason behind this increase is not clear. The increase appears to have happened in most units after 2006. Only a few units have pre-printed orders for the measurement of magnesium or the treatment of hypomagnesaemia. There does not appear to be any groundbreaking studies in the last 6 years to explain this change in administration of intravenous magnesium, except possibly in treatment of acute asthma [19].

## Conclusion

The measurement of serum magnesium and the use of intravenous magnesium in this tertiary care center have increased significantly. The reasons for this change in practice are unclear. More research is needed to determine whether this change in practice has any benefit on patient outcomes.

## Abbreviations

GFR: Glomerular filtration rate
MDRD: Modified Diet in Renal Disease
EKG: Electrocardiogram
